# Identification and Validation of Human Papillomavirus Encoded microRNAs

**DOI:** 10.1371/journal.pone.0070202

**Published:** 2013-07-30

**Authors:** Kui Qian, Tuuli Pietilä, Mikko Rönty, Frederic Michon, Mikko J. Frilander, Jarmo Ritari, Jussi Tarkkanen, Lars Paulín, Petri Auvinen, Eeva Auvinen

**Affiliations:** 1 Institute of Biotechnology, University of Helsinki, Helsinki, Finland; 2 Haartman Institute, Department of Virology, University of Helsinki, Helsinki, Finland; 3 Department of Virology and Immunology, Helsinki University Hospital Laboratory, Helsinki, Finland; 4 Department of Pathology, Helsinki University Hospital Laboratory, Helsinki, Finland; Instituto Butantan, Brazil

## Abstract

We report here identification and validation of the first papillomavirus encoded microRNAs expressed in human cervical lesions and cell lines. We established small RNA libraries from ten human papillomavirus associated cervical lesions including cancer and two human papillomavirus harboring cell lines. These libraries were sequenced using SOLiD 4 technology. We used the sequencing data to predict putative viral microRNAs and discovered nine putative papillomavirus encoded microRNAs. Validation was performed for five candidates, four of which were successfully validated by qPCR from cervical tissue samples and cell lines: two were encoded by HPV 16, one by HPV 38 and one by HPV 68. The expression of HPV 16 microRNAs was further confirmed by *in situ* hybridization, and colocalization with p16INK4A was established. Prediction of cellular target genes of HPV 16 encoded microRNAs suggests that they may play a role in cell cycle, immune functions, cell adhesion and migration, development, and cancer. Two putative viral target sites for the two validated HPV 16 miRNAs were mapped to the E5 gene, one in the E1 gene, two in the L1 gene and one in the LCR region. This is the first report to show that papillomaviruses encode their own microRNA species. Importantly, microRNAs were found in libraries established from human cervical disease and carcinoma cell lines, and their expression was confirmed in additional tissue samples. To our knowledge, this is also the first paper to use *in situ* hybridization to show the expression of a viral microRNA in human tissue.

## Introduction

Human papillomaviruses (HPV) preferentially infect keratinocytes of mucous membranes or skin and cause numerous benign and malignant lesions at different anatomical locations. HPV infection is the necessary cause of cervical cancer [1] and is associated with varying proportions of other cancers of the anogenital tract, head and neck region, and skin [2]. High-risk human papillomavirus types 16 and 18 are known to be associated with more than 70% of cervical cancers [3], [4]. Squamous cell carcinoma of the cervix develops through cervical intraepithelial neoplasia (CIN) grades 1–3. A proportion of all CIN grades may regress, but CIN3 is considered a precancer with potential to progress to cervical cancer. High-risk HPVs are also associated with adenocarcinoma *in situ* and adenocarcinoma of columnar epithelium.

Establishment of HPV infection requires the host cell to pass early cell cycle progression and enter M phase in the undifferentiated proliferating basal cell layer [5]. Progeny virus production occurs exclusively in differentiated suprabasal layers of the epithelium, and cannot take place if epithelial differentiation is disturbed. Regulation of papillomavirus replication and successful progeny virus production, or pathogenesis of HPV associated diseases is not completely understood. The environment of epithelial cells committed to differentiate is crucial and essentially involves regulatory changes in mRNA and microRNA expression.

MicroRNAs (miRNAs) are small, 19–24 nucleotide long noncoding RNAs that post-transcriptionally regulate messenger RNA (mRNA) expression. We have previously identified a number of cellular microRNAs regulated by the HPV 16 E5 oncogene [6]. Importantly, up-regulation of human miR-146a and down-regulation of human miR-203 and miR-324-5p, with subsequent regulation of their known and predicted target genes, was shown. Those results suggested that microRNAs play key roles in regulating adhesion and differentiation of epithelial cells, as well as attenuation of host immune response, which are crucial events involved in carcinogenesis [6].

The human genome encodes 1600 miRNAs listed in the miRBase [7]. A number of DNA viruses encode their own miRNAs as well. Most of the known viral miRNAs are found in herpesviruses [8], but also polyomaviruses [9], adenoviruses [10] and ascoviruses [11] encode their own miRNAs. Viral miRNAs are mostly generated from precursor miRNA (pre-miRNA) by Dicer cleavage and incorporated into the RNA-induced silencing complex (RISC), similar to host miRNAs [8]. Pre-miRNA can be generated by Drosha/Dicer cleavage of primary miRNAs (pri-miRNAs), mirtron, tRNase Z cleavage of tRNA-like pri-miRNA, or alternative folding of transfer RNAs [12] or small nucleolar RNAs [13]. Each pre-miRNA forms a hairpin structure, which encodes two products, one mature and one star miRNA (miRNA*). Typically star miRNA is expressed at lower level and is usually degraded [7]. Mature miRNAs almost always have variants, named isomiRs, which can also be functional miRNAs [14].

Viral miRNAs can target both viral and cellular mRNAs for down-regulation [15]. They contribute to cellular reprogramming by regulating the switch from latent to lytic viral infection, and by modulating the immune responses of the infected host [13]. Polyomavirus miRNAs target viral early transcripts to negatively regulate T antigen expression, and they also promote immune evasion by targeting cellular genes involved in host immune response, which subsequently leads to enhance viral replication [16]–[19]. The functional and molecular similarities among these double-stranded DNA (dsDNA) viruses causing long-term latent infections, especially simian virus 40 (SV40) [9], human polyomaviruses BKV and JCV [16], and BPCV [20], suggest that HPV could also encode viral miRNAs. To date, no studies have been able to validate viral miRNAs in papillomavirus infected cells using standard sequencing [21] or next generation sequencing techniques [22]. However, Gu et al. [23] recently presented a careful prediction of several microRNAs in mucosal and cutaneous HPVs from papillomavirus sequence data using well-established algorithms, and proposed putative HPV microRNAs with similarity to known human microRNAs. Despite considerable efforts by several authors, no validated papillomavirus miRNAs have been established so far. The lack of an efficient cell culture system to study viral replication in the context of epithelial differentiation and maturation has hindered miRNA discovery in HPV.

To study whether HPV replication and pathogenesis might be regulated by virally encoded miRNAs, we sequenced small RNA (sRNA) libraries derived from two HPV 16 immortalized cell lines, HPK IA and HPK II [24], and from ten formalin fixed paraffin embedded (FFPE) tissue samples from HPV infected cervical epithelium using SOLiD 4 technology. We used these data and miRSeqNovel software [25] to predict novel miRNAs and their likely pre-miRNAs. We further validated the candidate miRNAs in a number of tissue samples from HPV associated cervical disease and also in HPV 16 positive cell lines CaSki [26] and SiHa [27] by qPCR, and showed miRNA expression in cervical tissue by *in situ* hybridization. Biological functions of the predicted cellular and viral targets of HPV encoded microRNAs suggest similar activities to those of polyomavirus miRNAs, and propose a potentially important role in the progression of HPV infections.

## Materials and Methods

### Ethics Statement

The use of anonymized archival human samples in this study, without written informed consent from the donor or the next of kin, was approved by the Coordinating Ethical Committee of the Helsinki and Uusimaa Hospital District (69/E0/07) and the National Authority for Medicolegal affairs (2461/04/044/07).

### Cell Culture and Nucleic Acid Extraction from Cells

HPK IA and HPK II cells were established and provided by Dr. Matthias Dürst (German Cancer Research Center, Heidelberg, Germany; present address: University Clinic Jena, Germany) [24]. The cells were established by transfection of primary human foreskin keratinocytes with HPV 16. CaSki epidermoid cervical carcinoma cells and SiHa human cervical tumor cells were purchased from the American Type Culture Collection ATCC (Manassas, VA). All cells were cultured in DMEM (Sigma-Aldrich Inc., Saint Louis, MO) supplemented with 10% fetal bovine serum and penicillin/streptomycin at 37°C and 5% CO2 in a humidified incubator. Total RNA from cultured cells was isolated using the mirVana RNA isolation kit (Ambion, Austin, TX). RNA concentrations were measured in NanoDrop instrument (Thermo Scientific, Wilmington, DE). DNA was isolated from cells using the QIAamp DNA kit (Qiagen, Hilden, Germany).

### Paraffin-embedded Clinical Samples and Nucleic Acid Extraction

Altogether 27 anonymized FFPE cervical tissue samples representing normal squamous and columnar epithelium, CIN1-3, squamous cell carcinoma, adenocarcinoma *in situ*, and adenocarcinoma were obtained from the Department of Pathology, Helsinki University Hospital Laboratory. At the time of selection the HPV infection status of the samples was unknown. Total RNA and total DNA from altogether four 20-micrometer sections from FFPE tissue samples were harvested using the RecoverAll total RNA isolation kit (Ambion). RNA and DNA concentrations were measured in NanoDrop.

### SOLiD Sequencing Library Generation and RNA Sequencing

Small RNA fragments of ∼18–25 nt were enriched from total RNA preparations (ca. 6 micrograms) from HPK IA cells, HPK II cells and ten tissue samples by flashPAGE system (Ambion). All of the enriched small RNA fractions (30–60 ng) were used for preparing the libraries and subsequently ligated to adaptors in the SOLiD™ Total RNA-Seq kit (Ambion). The chemistry preferentially includes mature miRNA molecules and excludes RNA degradation products, double-stranded DNA, and single-stranded DNA molecules. Target RNA has to have intact phosphorus and hydroxyl groups at the ends. The adapter consists of DNA, which is partially single-stranded and this part hybridizes to target RNA. Ligation is performed with a specific RNA-DNA enzyme mixture. Next, small RNAs were reverse transcribed into cDNA libraries, size selected using PAGE gel, and amplified with PCR primers introducing barcode sequences. The libraries were prepared for emulsion PCR (emPCR) according to SOLiD sequencing instructions followed by sequencing using the SOLiD 4 instrument (Life Technologies, Carlsbad, CA).

### HPV Genotyping

HPV genotypes present in the tissue samples were determined using the universal ligation detection reaction (LDR) method which we recently applied for HPV genotyping in our laboratory, with slight modifications [28]. The method is based on type-specific multiplex PCR amplification followed by LDR and detection of fluorescent products on microarray platform. Human beta-globin was co-amplified in the assay to control for sample quality. The signals were detected using a GenePix Autoloader 4200AL laser scanning system (Axon Instruments, Foster City, CA) and GenePix program version 6.1 (Axon Instruments). Scanning data were analyzed as described previously [28]. For comparison, the presence of high-risk HPV DNA in the samples was analysed using the well-established Hybrid Capture 2 assay (Qiagen, Gaithersburg, MD). HPV genotyping was additionally performed by a Luminex based assay at Quattromed HTI (Tartu, Estonia), except for two samples which were inadequate for this analysis.

### PCR Confirmation of the miRNA-encoding Region

The presence of the miRNA-encoding region in human samples and in cell lines was confirmed by DNA PCR with specific primers (Table S1). A representative set of PCR products was sequenced using BigDye® Terminator v3.1 Cycle Sequencing Kit (Applied Biosystems, Foster City, CA), Mag-BindTM SeqDTRTM kit (OMEGA bio-tek, Norcross, GA), and ABI3130XL Genetic Analyzer (Applied Biosystems).

### Validation of Candidate Viral miRNAs by TaqMan RT-qPCR

TaqMan quantitative PCR assays were custom designed for seven candidate viral mature miRNAs (Life Technologies), two out of which failed in the design process. Reverse transcriptase reactions contained 13.3 ng/µl total RNA, 1× stem-loop RT primer, 1× RT buffer, 0.25 mM of each dNTP, 3.33 U/µl MultiScribe reverse transcriptase and 0.25 U/µl RNase inhibitor. The 15-µl or 7.5-µl reactions were incubated in thermocycler for 30 min at 16°C, 30 min at 42°C, 5 min at 85°C, and then held at 4°C.

Real-time PCR was performed using TaqMan Universal PCR Master Mix (No AmpErase UNG) kit protocol on a LightCycler 480 System (Roche Applied Science, Mannheim, Germany). The 10-µl PCR reactions included 2 µl RT product, 1× TaqMan Universal PCR Master Mix, 1× miRNA specific TaqMan small RNA Assay. The reactions were pipetted with Corbett CAS-1200 biorobot (Qiagen). The reactions were incubated in a 384-well plate at 95°C for 10 min, followed by 50 cycles of 95°C for 15 s and 60°C for 1 min, and then cooled at 40°C for 30 s. All reactions were run in triplicates. The size and the quality of RT-PCR products were defined in Bioanalyzer using DNA 1000 kit (Agilent, Waldbronn, Germany).

### *In situ* Hybridization

*In situ* hybridization was performed as described previously, with modifications [29]. Single (5′) or double (5′ and 3′) digoxigenin labeled miRCURY LNA detection probes (Exiqon) are described in Table S1. Specific probes for mature HPV16-miR-H1 and HPV16-miR-H2, together with a probe for hsa-miR-205 expressed in cervical tissue [30], snRNA U6 positive control probe, and a scramble negative control probe were used. An oligonucleotide probe for HPV 16 E1 mRNA, not hybridizing to the putative miRNA sequence, was used to confirm that *in situ* hybridization signal for HPV16-miR-H1 is obtained specifically from microRNA, not from mRNA, in our experimental conditions.

Briefly, FFPE tissues adhered to positively charged glass slides were deparaffinized, digested with 10–20 µg/ml proteinase K, treated with 0.2% glycine, and fixed in 4% PFA. After acetylation the slides were prehybridized in microRNA ISH Buffer (Exiqon) for 10–15 min at Tm-30°C and hybridized with 40 nM probe (1 nM for U6 probe) at Tm-30°C overnight. Hybridization signals were detected using alkaline phosphatase conjugated anti-DIG-Ab and NBT/BCIP color substrate.

### Immunohistochemical Staining

Hematoxylin-eosin staining to reveal tissue morphology, as well as immunohistochemical staining for p16INK4a were performed according to routine protocols for diagnostic samples.

### Bioinformatic Analysis of SOLiD Sequencing Data

The reads in SOLiD colorspace format were mapped to papillomavirus genomes and human genome using SOLiD small RNA pipeline (RNA2MAP) with default parameters. The human miRNA annotation was from miRBase V17 [7]. The viral reference genome was constructed by concatenating 393 complete papillomavirus genomes retrieved from NCBI, including known subtypes and isolates of HPV and papillomaviruses in other species (Table S2). The mapped results were converted to GFF files using MaToGff (Applied Biosystems). The SOLiD csfasta and QV_qual format raw data of mapped reads are publicly available at GEO, series record GSE42380.

### Prediction of Candidate Viral miRNAs

We predicted novel virus-encoded miRNA candidates from the small RNA sequencing data using our recently developed miRSeqNovel software [25]. The software provides convenience in adjusting the prediction parameters. Because of lacking HPV encoded miRNA annotation, we performed the prediction in two sets of parameters. In the first round, reads with counts less or equal to three in each library were not used in the next step. The remaining reads within 40 nt gap to other reads were combined to be considered as pre-miRNA candidates. Also, the regions 100 nt upstream or downstream of mapped reads were blasted to search for candidate pre-miRNA sequences (Figure S1). Candidates shown at least in two libraries were further studied for their structures. We selected the candidate miRNAs for further validation based on the first round of predictions.

In the second round, reads from all twelve libraries were pooled together. Reads with counts less or equal to two were discarded. Subsequent steps were performed as in the first run, except that pre-miRNAs were extended to cover the reads overlapping with the predicted region. R codes used for prediction are available on request.

### Prediction of Novel Viral miRNA Targets

Human target genes of novel viral miRNAs were predicted using TargetScan custom miRNA prediction methods [31]. Putative targets within the viral genome were predicted using TargetScan Perl script.

## Results

### Mapping SOLiD Sequencing Reads to Virus Genomes

In this work we have applied deep sequencing technology to profile small RNA expression from the HPV genome in HPV containing cell lines and human cervical tissues. Libraries from small RNA fractions of two cell lines HPK IA and HPK II [24] as well as ten tissue samples were sequenced using SOLiD 4 platform. The number of total reads in the different libraries varied from 4.5 million to 97 million (Table 1). The viral reference genome for mapping the sequencing reads was constructed by concatenating together all available nucleotide sequences of papillomavirus types and isolates in the NCBI database (Jan 2011, 393 complete genomes, Table S2). Obtained reads were mapped to the papillomavirus reference genome to specifically find reads aligned to papillomavirus sequences. No similarities with known human microRNA sequences were found. Full-length sequences of the predicted mature viral miRNAs, except for HPV16-miR-H6-1 which we did not attempt to validate, did not map to the official human genome GRC37. IsomiRs of HPV16-miR-H6-1 or other reads in HPV16-miR-H6 did not match to the human genome. We are thus convinced that our proposed miRNA sequences represent virally encoded microRNAs. The number of total reads mapped to the papillomavirus reference genome ranged from 61473 to 1.4 million, roughly 1∼2% in each library. Mapping of reads to the human genome show that around 50% of total reads mapped to known human miRNA, and around 10% mapped to the human genome (Table 1). Although a somewhat lower proportion of reads mapped to known miRNAs in Lib11 and Lib12 than in other libraries, the sequencing library preparation and mapping were considered successful.

**Table 1:** Mapped results of the twelve small RNA sequencing libraries.

Library	Total reads number	Mapped reads number(% of total reads)	Library description	% of total readsmapped tohuman miRNAs	% of total reads mapped to human genome
Lib1	7120921	136685 (1.92%)	HPK IA	56.38%	7.43%
Lib2	63395143	552214 (0.87%)	HPK II	47.61%	9.91%
Lib3	4511000	61473 (1.36%)	normal cervical squamous epithelium	41.64%	8.28%
Lib4	35760385	950230 (2.66%)	adenocarcinoma *in situ*	51.81%	7.99%
Lib5	18188245	275672 (1.52%)	squamous cell carcinoma	43.34%	7.67%
Lib6	36598210	922368 (2.52%)	adenocarcinoma *in situ*	53.66%	9.87%
Lib7	50597264	637917 (1.26%)	CIN1/condyloma	51.07%	10.60%
Lib8	91674354	1187602 (1.30%)	CIN1/condyloma	42.20%	7.80%
Lib9	21433903	330383 (1.54%)	CIN2	51.40%	6.82%
Lib10	21310108	411991 (1.93%)	CIN2	53.19%	9.43%
Lib11	62194515	1084089 (1.74%)	CIN2	33.69%	9.54%
Lib12	97534820	1446426 (1.48%)	normal cervical columnar epithelium	16.38%	16.76%

For each library, total reads from SOLiD small RNA sequencing and reads mapped to the papillomavirus reference genome are presented. Names of cell lines or histology of tissue samples are given in library description. Percentage of reads mapped to human miRNAs and to the human genome, respectively, are given. CIN, cervical intraepithelial neoplasia.

### Prediction of Novel Viral miRNA Candidates

The mapping data from SOLiD sequencing were used to predict novel viral candidate miRNAs. Prediction of novel viral microRNAs was performed by mirSeqNovel [25] which revealed several candidate sequences (Table S3–S6). The pre-miRNA sequences were named following the annotation instructions in miRBase [7], such as HPV16-miR-H1 for pre-miRNA and HPV16-miR-H1-1 for mature miRNA. We performed two rounds of predictions regarding whether the pre-miRNA should be extended to cover low count reads (see Materials and Methods). Comparison of the results from the first and secondary prediction rounds revealed advantages and disadvantages in both rounds. When star miRNA, which was estimated by second highest counts, is not fully covered by the pre-miRNA in the first round, the second round will give a better prediction of pre-miRNA. However, in the second round, some low expression reads with counts less than three, which would possibly represent noise, may add additional nucleotides to pre-miRNA, leading to suboptimal RNA secondary structure. We thus considered both results but focused on the first round for validation of candidate miRNAs.

Putative HPV miRNAs for further studies were selected based on: (1) clinical relevance of HPV types, particularly HPV 16 encoded candidates; (2) Candidates encoded by high risk and low risk mucosal HPV types; (3) Candidates encoded by skin HPV types.

Sequencing results showed strong evidence of six novel HPV encoded microRNA candidates in the first prediction round (Table 2). However, given the high background and low expression possibly leading to false-positive predictions, in the second prediction round we combined all the libraries together to obtain a library containing all sequenced reads, which enabled more reasonable prediction and easier finding of isomiRs. By further diminishing background we were able to identify additional HPV 16 encoded putative miRNAs, but, using this approach, HPV16-miR-H2 found in the first round was excluded due to suboptimal structure of extended pre-miRNA to cover the background reads. HPV16-miR-H1 was shown using both strategies. We finally combined the results and established a total of nine novel putative HPV encoded miRNAs (Table 2, Table S7, Figure S2, Figure S3). None of these putative miRNA sequences had similarity to known human microRNAs.

**Table 2:** Predicted viral miRNA candidate.

miRNA name	Reference genome	Location	Read counts	Annotation	Strand	Mature sequence
HPV6-miR-H1	NC_001355.1	1828–1716	1828	E1	–	TGGTTTTCAGGTATATTTAA
HPV16-miR-H1	NC_001526.2	2635–2716	45	E1	+	AGTGTATGAGCTTAATGATAA
HPV16-miR-H2	FJ610147.1	56-1/7906–7851	1203	LCR	–	ATGTGTAACCCAAAACGGTTTG
HPV16-miR-H3^§^	NC_001526.2	518–642	39	LCR	+	CAACTGATCTCTACTGTTA
HPV16-miR-H5^§^	NC_001526.2	2471–2556	46	E1	+	GTAAAGCATAGACCATTG
HPV16-miR-H6^§^	NC_001526.2	6684–6584	6161	L1	–	ATCAACAACAGTAACAAA
HPV38-miR-H1	U31787.1	724–621	67	E7	–	ATCACGAAGAGTAGCTTG
HPV45-miR-H1	EF202157.1	6676–6790	282	L1	+	AGTATAGTAGACATGTGGAGGA
HPV68-miR-H1	GQ472851.1	6210–6305	274	L1	+	ACAAATGTCTGCAGATGTCTA

Each row presents one candidate miRNA with name, reference genome, pre-miRNA location in the genome, total read counts of pre-miRNA, viral gene annotation in corresponding region, strand information and mature miRNA sequence. Some miRNAs were shown in more than one isolate/subtype papillomavirus genomes. ^§^ Prediction results from second round.

**Figure 1:**
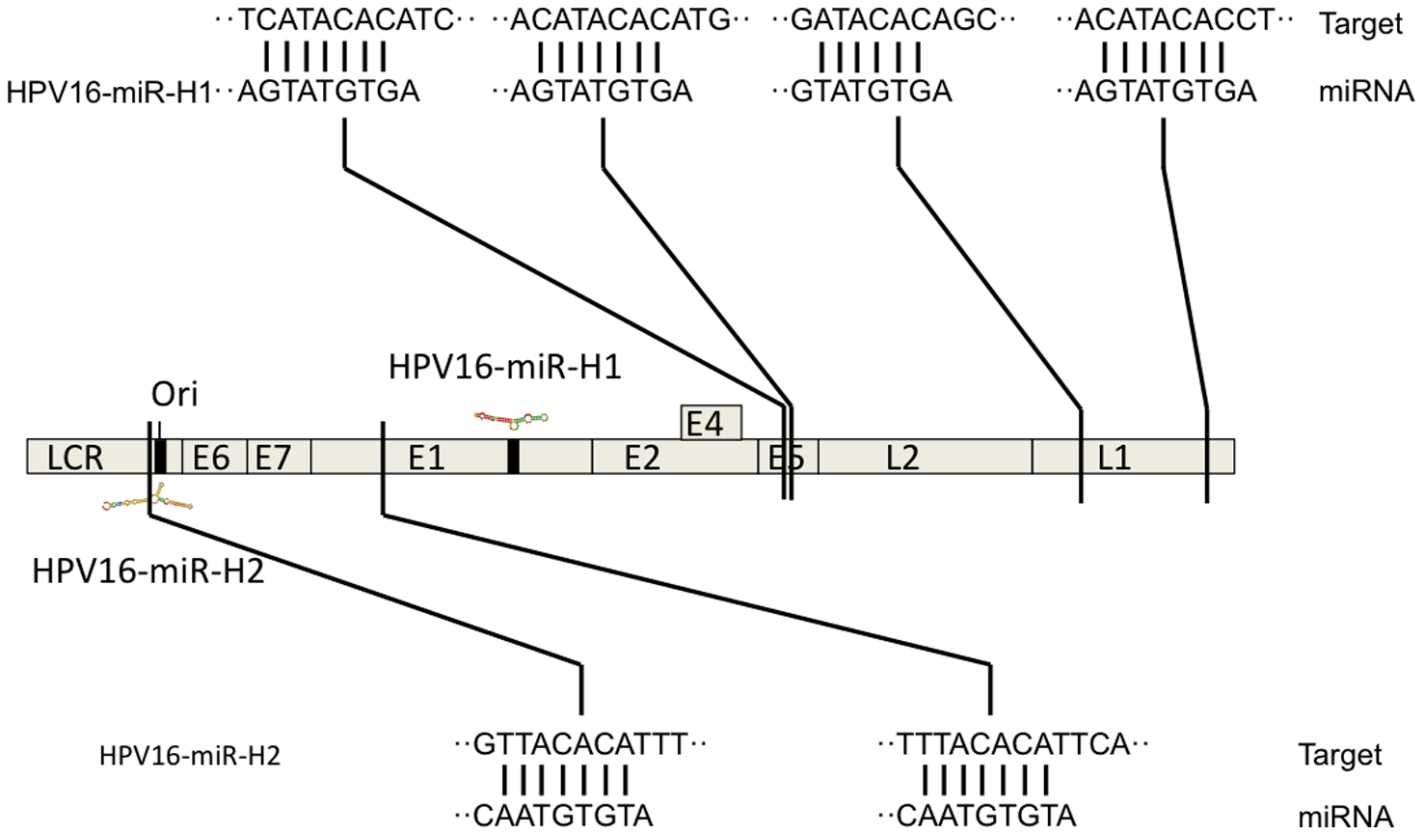
Locations and putative target sites of HPV 16 encoded microRNAs. Locations of HPV16-miR-H1-1 and HPV16-miR-H2-1 in the viral genome are shown as black bars and the predicted secondary structures are given next to the bars. For each miRNA, the seed sequences and predicted target sequences in the HPV genome are shown.

Five of the selected candidates were encoded by the clinically most relevant HPV type, HPV 16. Two of the candidates were studied in more detail: HPV16-miR-H1, encoded by a region within the E1 gene on the positive DNA strand, and HPV16-miR-H2, encoded by the negative strand complementary to the long control region (LCR) (Figure 1). Interestingly, HPV16-miR-H2 coding sequence was found in HPV 16 isolates, but there is a one-nucleotide deletion in the mature miRNA sequence in the prototype HPV 16 genome (NC_001526.2). We compared HPV16-miR-H2 against all HPV 16 sequences in the NCBI database (April 26, 2013), and altogether 267 out of 329 sequences gave a perfect match to HPV16-miR-H2, suggesting that this sequence represents the major form in HPV 16.

HPV16-miR-H3 is localized in the LCR, HPV16-miR-H5 in E1 coding region and HPV16-miR-H6 in the antisense strand complementary to L1. HPV6-miR-H1 is localized in the antisense strand of HPV 6 E1 region. HPV38-miR-H1 coding sequence resides in E7 region. The coding sequences for HPV45-miR-H1 and HPV68-miR-H1 are localized in the corresponding L1 coding regions.

The mature HPV16-miR-H1-1 miRNA is located in the loop region of HPV16-miR-H1 pre-miRNA as suggested by miRSeqNovel prediction (Table S7 and Figure S4). Other isomiRs in HPV16-miR-H1 are also found in the loop region. Because of low read counts in sequencing data, it was difficult to distinguish mature miRNA and star miRNA from RNA degradation background. Because of the background, we further checked whether the predicted miRNA is correct by screening this with VMir [9], and found that the mature HPV16-miR-H1-1 miRNA, not the isomiRs, is located in the arm region of a shorter precursor sequence (Figure S4).

### Validation of Putative HPV Encoded miRNAs by TaqMan qPCR

We selected altogether seven HPV miRNA candidates from both prediction rounds for validation based on clinical relevance, quality of the reads and computer assisted structural features (Figure S2). Validation was performed using tailored TaqMan MicroRNA Assays for putative mature miRNAs (Table 3, Table S8). Two of the seven selected miRNAs (HPV45-miR-H1-1, and HPV16-miR-H6-1) failed at assay design and thus validation was performed for five putative miRNAs.

**Table 3:** Summary of TaqMan miRNA qPCR, DNA PCR and p16 staining results, as well HPV detection and/or genotyping results by LDR, Luminex and Hybrid Capture 2.

		TaqMan qPCR positive results/total reactions performed						DNA PCR					
Sample		HPV6-miR-H1	HPV16-miR-H1	HPV16-miR-H2	HPV38-miR-H1	HPV68-miR-H1	U6	HPV16-miR-H1	HPV16-miR-H2	p16	LDRgenotyping*	Luminexgenotyping*	HC2
SiHa		0/9	4/6	0/6	3/6	3/6	6/6	+	+	NA	NA	NA	NA
CaSki		0/9	4/6	0/6	3/6	1/6	6/6	+	+	NA	NA	NA	NA
HPK IA		0/9	3/6	3/6	3/6	6/6	6/6	+	+	NA	NA	NA	NA
HPK II		0/9	3/6	0/6	2/6	2/6	6/6	+	+	NA	NA	NA	NA
89	CIN1/Cond pl	0/9	0/9	0/9	4/9	2/9	6/6	–	–	+	16, 18	33, 58	+
79	CIN1/Cond pl	0/9	1/9	0/9	5/9	3/9	6/6	–	–	+	16, 58	16, 33, 58	+
100	CIN2	0/9	0/9	0/9	1/9	1/9	6/6	–	–	+	–	–	–
98	CIN2	0/9	1/9	0/9	2/9	3/9	6/6	–	–	+	16	16	–
99	CIN2	0/9	1/9	0/9	4/9	2/9	6/6	–	–	+	16, 52	16, 35, 52, 56	+
53	CIN3	0/9	2/9	0/9	4/9	1/9	6/6	–	–	+	16, 18, 33	ND	+
49	CIN3	0/9	2/9	1/9	4/9	6/9	6/6	+	–	+	16	16, 31, 35	–
18	CIN3	0/9	2/9	1/9	5/9	5/9	6/6	–	–	+	6, 16	ND	–
41	CIN3	0/9	6/9	1/9	7/9	4/9	9/9	+	+	+	16, 18	16	–
48	SCC	0/9	3/9	2/9	5/9	3/9	6/6	+	+	+	16, 18	16	+
8	SCC	0/9	5/9	1/9	2/9	5/9	6/6	+	+	+	16, 18	16	+
102	SCC	0/9	9/9	4/9	5/9	4/9	9/9	+	+	+	16	16	+
87	AIS	0/9	1/9	0/9	2/9	2/9	6/6	–	–	+	16, 58	16, 18, 58	–
97	AIS	0/9	1/9	1/9	1/9	3/9	6/6	+	+	+	16, 18	16	+
76	AIS	0/9	2/9	0/9	2/9	4/9	6/6	–	–	+	16	45	–
27	AIS	0/9	6/9	4/9	2/9	3/9	6/6	+	+	+	16, 18	16	+
47	AC	0/9	2/9	1/9	3/9	6/9	6/6	–	–	+	16	–	+
103	Normal SE	0/9	3/9	2/9	3/9	1/9	6/6	–	–	–	16	–	–
101	Normal CE	0/9	0/9	0/9	1/9	1/9	9/9	–	–	–	16, 18	16	–

Obtained positive results in TaqMan qPCR out of performed runs are given. HPV6-miR-H1 did not give any positive results. Positive signals were obtained for HPV16-miR-H1, HPV16-miR-H2, HPV38-miR-H1 and HPV68-miR-H1 from cell lines and patient samples. Positive control U6 gives positive result in every reaction. The presence of pre-miRNA coding regions for HPV16-miR-H1 and HPV16-miR-H2 was confirmed by DNA PCR in all cell lines and many tissue samples. Results of p16 tissue staining, as well as results of HPV genotyping by LDR or Luminex, and high risk HPV detection by Hybrid Capture 2 are given. NA, not analyzed. ND, not done (inadequate sample). CIN1-3, cervical intraepithelial neoplasia 1–3; Cond pl, condyloma planum; SCC, squamous cell carcinoma; AIS, adenocarcinoma *in situ*; AC, adenocarcinoma; SE, squamous epithelium; CE, columnar epithelium; LDR, ligase detection reaction based HPV genotyping assay; Luminex, Luminex based HPV genotyping assay; HC2, Hybrid Capture 2 HPV detection assay; *, numbers are HPV types; NA, not analyzed; ND, no data (not enough material for testing).

We were able to validate the expression of four out of five miRNAs in cell lines or cervical tissues. Altogether, the presence of miRNAs was analyzed in SiHa, CaSki, HPK IA, HPK II cell lines and 19 tissue samples (Table 3). All microRNAs were detected at high cycle counts in TaqMan qPCR, suggesting fairly low expression levels in RNA preparations prepared from tissue samples where only a subset of cells represent tumor tissue. The size and the quality of representative qPCR products were additionally confirmed in Bioanalyzer. HPV16-miR-H1-1 was detected in all cell lines and 16 tissue samples (Table 3, Table S8). Weak signals for HPV16-miR-H2-1 were shown by TaqMan qRT-PCR in HPK IA cells and in ten tissue samples. High cycle of threshold (Ct) values were obtained for HPV68-miR-H1-1 in tissue samples and in cell lines. However, Bioanalyzer analysis showed some background, suggesting additional primer binding. Similar results were obtained for HPV38-miR-H1. Finally, although sequencing results suggested high level expression of HPV6-miR-H1 (Table S7, Figure S2A), it could not be validated in qRT-PCR. The U6 positive control showed strong signals in all the runs confirming good technical performance and good quality of the samples.

Typically, different methods to study miRNA expression may produce different results, and they are not quantitatively comparable. In the present study, miRNA sequencing data showed hundred-fold higher read counts for HPV16-miR-H2 than for HPV16-miR-H1. However, in qRT-PCR validation the signal for HPV16-miR-H2-1 was weaker than for HPV16-miR-H1-1. Similarly, HPV6-miR-H1 showed high read counts in sequencing but could not be validated by qRT-PCR (Table 3, Table S7, Table S8). The most likely explanation for these discrepancies is that the RNA ligase used in the library construction has different preferences for the different nucleotides, as has been suggested earlier [12], [32]. The method of library construction should strongly prefer microRNA to RNA degradation products or DNA [33]. Further analysis will be required to validate whether HPV6-miR-H1-1 is indeed a functional microRNA.

### Presence of miRNA Encoding Regions and Genotyping of HPV

We further confirmed the presence of the predicted HPV16-miR-H1-1 and HPV16-miR-H2-1 coding regions by PCR amplification of the relevant HPV genomic regions followed by Sanger sequencing for a representative set of samples in cell lines and several tissue samples (Table 3). No amplification products were obtained for HPV 6, HPV 38 and HPV 68 pre-miRNA coding regions.

HPV genotypes in the clinical samples were determined using an assay recently developed by us [28]. As expected, HPV types 16 and 18 were the most frequently occurring genotypes, and several samples harbored two HPV types (Table 3). HPV 16 was found in all cervical lesions, which was somewhat surprising even though it is expected to be the most prevalent HPV type in cervical neoplasia. All negative controls included in the assay remained negative. We have shown that the LDR assay developed previously by us and used for genotyping in this work may be more sensitive than commercial HPV assays [28], which may explain the high detection rate. HPV 38 was not included in the genotyping assay because the assay was originally designed for mucosal HPV types. All except two samples were further studied using a Luminex based genotyping assay. Similar results were obtained, although different genotypes were found in two samples, and two LDR positive samples remained negative in the Luminex assay. Altogether eight LDR positive samples remained negative in Hybrid Capture 2 liquid hybridization assay for high-risk HPV, which suggests higher sensitivity of our LDR assay as compared to Hybrid Capture 2, as we have proposed earlier [28].

### Expression of HPV miRNAs in Cell Lines and Tissue Samples

We studied the expression of HPV16-miR-H1-1 and HPV16-miR-H2-1 in cell lines and tissue samples. Typically HPV infected cell populations are not seen throughout tissue samples but in restricted areas. We conducted *in situ* hybridization for HPV16-miR-H1-1 and HPV16-miR-H2-1 in those samples which had been used for sequencing libraries, as well as 14 additional samples including one sample containing normal columnar and squamous tissue. Interpretation of *in situ* hybridization results is presented in Table 4.

**Table 4:** Summary of miRNA *in situ* hybridization (ISH), DNA PCR and p16 staining results.

Sample		HPV16-miR-H1-1 ISH	HPV16-miR-H2-1 ISH	DNA PCR HPV16-miRs H1/H2	p16
9	CIN1	+	–	NA	+
10	CIN1	+	–	NA	+
28	CIN1	+	–	NA	+
39	CIN1	+	–	NA	+
40	CIN1	+	–	NA	+
95	CIN2	+	–	NA	+
96	CIN2	+	–	NA	+
100	CIN2	+	–	–	+
41	CIN3	+	–	+	+
49	CIN3	+	–	–	+
8	SCC	+	–	+	+
48	SCC	+	–	+	+
102	SCC	+	+	+	+
27	AIS	+	–	+	+
97	AIS	+	–	+	+
47	AC	+	–	–	+
104	Normal SE/CE	–	–	NA	–

Expression of HPV16-miR-H1-1 was shown in all disease tissues. Expression of HPV16-miR-H2-1 was shown in only one carcinoma sample 102. CIN1-3, cervical intraepithelial neoplasia 1-3; SCC, squamous cell carcinoma; AIS, adenocarcinoma *in situ*; AC, adenocarcinoma; SE, squamous epithelium; CE, columnar epithelium. NA, not analyzed.

**Figure 2:**
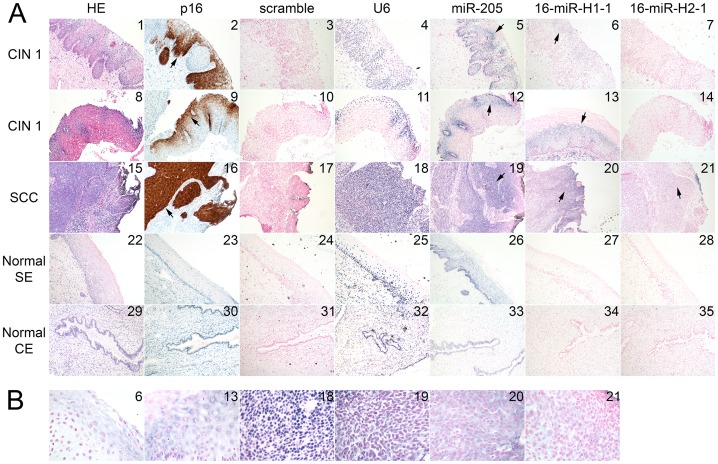
*In situ* hybridization for HPV16-miR-H1-1 and HPV16-miR-H2-1. (A) Hematoxylin-eoxin (HE) staining, immunohistochemical staining for p16, and *in situ* (italics) hybridization for scramble (negative control), U6 (positive control), human miR-205 (positive control for cervical tissue), HPV16-miR-H1-1 (16-miR-H1-1) and HPV16-miR-H2-1 (16-miR-H2-1). Shown are two cervical intraepithelial neoplasia grade 1 (CIN 1) samples (samples 10 and 28), one squamous cell carcinoma (SCC) (sample 102), as well as normal squamous epithelium (SE) (sample 104) and normal columnar epithelium (CE) (sample 104). Arrows point to positive signals. (B) Areas of selected picture fields shown in higher magnification to depict localization and positive hybridization signal. Same numbering is used as in (A).

Strong cytoplasmic signals for HPV16-miR-H1-1 were detected in several tissue samples, often colocalizing with p16INK4A (p16) immunohistochemical staining (Figure 2, fields 2, 9, 16 for p16; fields 6, 13, 20 for HPV16-miR-H1). p16 is a surrogate marker for high risk HPV. The encoding region for HPV16-miR-H1 is located within the E1 gene. MicroRNA specificity of HPV16-miR-H1 signals in our experimental conditions was confirmed by the absence of signal with a probe specific for E1 mRNA in altogether 10 tissue samples (an example is shown in Figure S5). Samples used for *in situ* hybridization had been fixed and paraffin embedded according to established routine protocols. Similar samples have been widely used for *in situ* hybridization for mRNA by us and by others [34]–[36]. Although some mRNA degradation in FFPE samples may take place, oligonucleotide probes can be expected to detect mRNA equally well as microRNA in conditions used in *in situ* hybridization. Further, the signal for HPV16-miR-H1-1 was seen in the cytoplasm, whereas strong nuclear signal for E1 mRNA has been reported previously [36]. Specificity of our hybridization conditions and our hybridization probe for HPV16-miR-H1-1 was thus confirmed by both negative signals with the E1 mRNA probe in these conditions, and by different localization of miRNA signal from previously published localization for E1 mRNA [36]. Low level expression of HPV16-miR-H2-1 was detected in one squamous cell carcinoma tissue (Figure 2, field 21).

U6, the positive control for hybridization, was detected in all samples. It also served as a control for localization accuracy as it resides in the nucleus, whereas HPV miRNAs are expressed in the cytoplasm. For negative control, we used a scramble probe that did not give any signal in HPV positive or HPV negative tissues. Human miR-205 was used as a positive control for miRNA expressed in cervical epithelium. Interestingly, the expression of human miR-205 was more intense and more broadly distributed in disease tissues as compared to healthy tissues (Figure 2, fields 5, 12, 19).

### Prediction of Viral miRNA Targets

We searched for the targets of HPV16-miR-H1-1 and HPV16-miR-H2-1 by TargetScan. Both of these miRNAs have a unique target fingerprint of 137 and 176 genes, respectively, in the human genome (Figure S6, Table S9). Interestingly they share 15 predicted targets: CDC2L6, EIF2C1, IMPAD1, BNC2, SNX27, TNRC6B, BACH2, CYP26B1, DDX19B, FGF7, PBRM1, PHACTR2, RBM3, RGS7BP, TEAD1.

Prediction of target sequences in the HPV 16 genome identified four target sequences for HPV16-miR-H1-1, two of which in the E5 gene and two in the L1 gene. HPV16-miR-H2-1 had two targets in the viral genome, one in the LCR region and another located in the L1 gene (Figure 1).

## Discussion

The association between human papillomavirus infection and the development of epithelial lesions is complex. Close to 200 HPV types have been characterized, and particularly the alpha HPV types are classified into high risk or low risk types according to their association with anogenital malignancies [37]. An individual can be infected with multiple HPV types, which may also increase the risk of developing a cervical lesion [38]. Moreover, many HPVs have been identified from healthy individuals without any clinical symptoms. The rare path from initial infection to severe epithelial lesion is still not understood in detail. We have previously shown that the viral E5 oncogene regulates the expression of a number of cellular mRNAs and microRNAs with key functions in cell adhesion and motility, epithelial differentiation, and immune response [6], [39], and we were able to confirm some of these regulations in cervical disease [40]. Our recent results suggest that microRNAs play a key role in the manifestation of HPV infections in epithelial target cells [6]. Many dsDNA viruses, such as polyomaviruses, encode miRNAs [9], [16], [20]. Papillomaviruses have initially been suspected to encode their own microRNAs because they have dsDNA genomes, they replicate mainly in the nucleus, and they have the ability to establish persistent infection and latency, but until now no papillomavirus miRNAs have been validated. Gu et al. previously presented a prediction of virally encoded microRNAs with homology to known human microRNAs based on bioinformatics analysis of papillomavirus sequences [23]. Here we present the first report on identification and validation of putative papillomavirus encoded microRNAs based on small RNA sequencing approach using libraries constructed from cultured cells and tissue samples.

Using SOLiD deep sequencing of small RNA libraries, we were able to identify altogether nine putative HPV encoded miRNAs in human cervical lesions and in HPV 16 transfected cell lines. Importantly, our strategy mainly identifies miRNAs that are expressed in persistent infection and may thus be associated with the development or maintenance of epithelial lesions. In contrast, putative viral miRNAs needed for productive replication of papillomaviruses may remain undiscovered, although two of the libraries used for small RNA sequencing were established from CIN1/condyloma, where productive viral infection may be ongoing. Conventional experimental infection to specifically search for microRNAs expressed in productive infection is not feasible with papillomaviruses, which do not replicate in laboratory *in vitro* conditions. In experimental settings allowing differentiation of epithelial cells, complete HPV life cycle with virion production has been reproduced [41], [42]. Such settings would provide a valuable tool to study the expression and role of microRNAs in virus replication.

Current understanding of human miRNA features was applied in screening for candidate genes of HPV miRNAs using miRSeqNovel software [25]. Accumulating miRNA sequencing data continuously serves to correct miRNA annotations in the miRBase [7]. We considered the relative sequence abundance as one of the main criteria in prediction of mature miRNAs [43]. Many candidate HPV microRNAs had low read counts, which made prediction of the precise features of novel microRNAs as well as annotation of isomiRs and star miRNAs challenging. Consequently some of our candidate miRNAs were not located in a typical mature miRNA location (Figure S2). While miRSeqNovel is useful in finding novel miRNA candidates when read counts are sufficient, accurate prediction of pre-miRNA is difficult when viral miRNAs are expressed at low levels and the background noise is relatively high. Therefore, more reads from tissue RNA, which may be limited when using FFPE samples, would be needed to obtain precise information about star miRNAs and isomiRs. This problem also calls for the development of highly efficient laboratory models of HPV infection.

Altogether, five of the putative papillomavirus microRNAs were encoded by HPV 16, one by HPV 38, one by HPV 68, one by HPV 45 and one by HPV 6. HPV 6, 16, 45 and 68 belong to alpha-papillomaviruses, whereas HPV 38 is a beta-papillomavirus. None of the candidate HPV encoded microRNAs had similarity to known human microRNA sequences. Of the validated microRNAs, HPV16-miR-H1-1 is located within the E1 region of the coding strand, and HPV16-miR-H2-1 in the negative strand corresponding to the LCR region. Intriguingly, the HPV16-miR-H2-1 sequence is present in a number of HPV 16 isolates but has a one-nucleotide deletion in the prototype sequence. Many of the isolates have been cloned from carcinoma tissues, suggesting that the ability to express this particular microRNA might promote carcinogenesis. Interestingly, the pre-microRNA sequence of HPV38-miR-H1, encoded by the E7 region, is shared by HPV types 22, 23, 120, 104 and 115, which are all members of the beta-papillomavirus genus. Moreover, the pre-miRNA sequence of HPV45-miR-H1, encoded by the L1 region, is partially similar to HPV 16, suggesting evolutionary divergence of viral miRNA function between HPV types. Although the deep sequencing read counts for the HPV 6 encoded miRNA were high, we were not able to validate it by qPCR, possibly due to the specific design of TaqMan assays. Because of the very short length of the miRNA there are very limited possibilities to alter the assay design if no results are obtained with the qPCR test.

Several of the putative miRNA sequences were encoded by the negative DNA strand, which disagrees with the consensus that all papillomavirus transcripts originate from the positive strand of papillomavirus genomes. Although in this first report we did not study the mechanisms of transcription of HPV microRNAs, our methodology should practically exclude RNA degradation products or DNA from sequencing libraries.

Viral miRNAs may also possess features that do not follow the canonical properties of human miRNAs [12]. The precursor sequence of HPV16-miR-H1 is still uncertain and needs further validation of length and exact sequence. Due to low expression level of this miRNA we were not able to establish its exact length. Merkel cell polyomavirus encoded MCV-miR-M1-5p, which was first predicted from VMir and validated [17], and further identified by Illumina sequencing and validated by qRT-PCR, has a 5′end 2-nt shift from the VMir predicted MCV-mir-M1 mature sequence, which has also been shown to exist and be functional in vitro [19]. Further studies are needed to prove whether the isomiRs presented here could also exist and be functional under some conditions.

Entire tissue samples consisting of both healthy and infected cells were used for these studies. Robust signals were seen in cervical tissue in *in situ* hybridization, often colocalizing with and restricted to regions staining for p16INK4a, which is considered a surrogate marker for high-risk HPV oncogene activity. *In situ* hybridization also showed altered distribution of human miR-205, whose high expression has been reported before in CaSki cells and in cervical cancer tissues [44]. miR-205 was also recently shown to promote proliferation of human cervical cancer cells [45]. Although some viral miRNAs are occasionally expressed at high levels, low level of expression has also been shown biologically relevant, for example for Merkel cell polyomavirus miRNA [19]. Those authors speculated that even low levels of viral miRNA expression might be sufficient to regulate host immune response [19]. However, the signals in the *in situ* assays for the U6, miR-205 and HPV miRNAs cannot be directly compared as a measure of the expression level.

Cellular targets of HPV encoded miRNAs give an overview of their putative functions. Gene ontology classes of the predicted cellular targets of HPV16-miR-H1-1 suggest important roles in host cell interactions of HPV 16, such as the cell cycle process (CUL3, CYP26B1, MAP3K11, PBRM1, SMC1A), especially the M phase (CYP26B1, PBRM1, SMC1A), which is important for viral infection [5]. A set of predicted target genes is involved in regulation of immune functions of the host, such as T cell activation (BCL11A, CHD7, ITGAM, RAG1) and immune system development (BCL11A, CHD7, RAG1, TCEA1). Involvement of this particular microRNA in neoplastic development is suggested by its putative target genes with key roles in focal adhesion (CAV2, IGF1R, ITGB8, PTEN, PIK3CD) and cell migration (CAV2, ITGAM, PAX6, PTEN, SEMA3F, ULK1). Importantly, target genes involved in epithelium development (RGMA, SHANK3, PAX6, PFN1, WNT4) and cancer (CBL, CYCS, FGF7, IGF1R, PTEN, PIK3CD, WNT4) address to a further role in the onset of epithelial abnormalities and oncogenesis. PIK3CD, involved in activation of cell growth, survival, proliferation and motility, in regulation of cell morphology, and in mediating host immune responses, is of particular interest, because the same target gene has been predicted for Merkel cell polyomavirus miRNA [19]. Further, we have previously reported activation of the Akt/PI3K pathway by the HPV 16 E5 oncogene [39]. The present results suggest that HPV encoded miRNAs may be involved in this process, taken into account that HPV16-miR-H1-1 has two putative target sites within the E5 gene.

Similar pathways are represented among the predicted targets of HPV16-miR-H2-1. These involve cell cycle process (SETD8, CYP26B1, FOXN3, HMGA2, MAP9, PAFAH1B1, PBRM1, TP53INP1, VASH1) and M phase (SETD8, CYP26B1, HMGA2, MAP9, PAFAH1B1, PBRM1), as well as immune regulation such as T cell activation (PKNOX1, SP3, XRCC4) and immune system development (JAK2, PKNOX1, SP3, XRCC4, FOXP1). Importantly, predicted target gene functions in cell migration (CDK5R1, ITGA5, PAFAH1B1, SRF) and cell adhesion (ARF6, FAT3, CHL1, COL19A1, CNTNAP2, CDK5R1, FLRT2, ITGA5, NLGN1, PCDH18, PCDHA family, SORBS1) suggest a possible involvement or interplay with the E5 viral oncogene, which showed very similar functions in our earlier studies [6], [39].

Both HPV 16 encoded microRNAs have two interesting common targets involved in cell cycle regulation. CYP26B1 encodes a CYP450 family enzyme crucial in retinoic acid metabolism, specifically the inactivation of all-trans retinoic acid (RA) and generation of its hydroxylated forms [46]. RA has been shown to regulate epithelial cell differentiation and inhibit the growth of HPV 18 harboring HeLa cervical carcinoma cells [47]. RA has also been shown to down-regulate the mechanisms protecting HPV harboring CaSki and HeLa cells from Fas/FasL mediated apoptosis [48]. Down-regulation of RA by HPV encoded miRNA through CYP450 would thus lead to increased resistance of HPV infected cells to apoptosis and stimulate cell growth. Another common target of HPV 16 encoded miRNAs, PBRM1, encodes polybromo-1, which can function as a transcriptional activator or suppressor of a number of genes by chromatin remodeling [49]. Its role as a negative regulator of cell proliferation could be counteracted by HPV 16 miRNA to allow expansion of the HPV infected cell population.

Other common target genes include fibroblast FGF7, encoding fibroblast growth factor 7, also known as keratinocyte growth factor (KGF). KGF is a potent epithelial growth factor, which has been implicated in epithelial morphogenesis in wound healing, and has been shown to be a bifunctional regulator of the growth of HPV 16 immortalized cervical epithelial cells [50]. Another common target gene, TEAD1 (or SV40 transcriptional enhancer factor SV40 transcriptional enhancer factor), encodes transcriptional enhancer factor 1 (TEF-1), which regulates cell proliferation, migration and epithelial-mesenchymal transition (EMT) induction, and has been shown to bind and activate the early HPV 16 promoter [51], [52].

Viral miRNAs have potentially evolved to provide ideal tools for viruses to modulate both viral and cellular gene expression. Viral miRNAs of SV40 [9], Merkel cell polyomavirus (MCV) [17], [19], JC virus and BK virus [16], and BPCV [20] share similar functions in negatively regulating viral early gene expression by targeting early transcripts (T-antigen), with subsequent escape from host immune attack and facilitation of viral replication. Despite the lack of sequence similarities, HPV has similar genome size and similar gene functions to those of polyomaviruses, which suggests that HPV might encode microRNAs with related functions. Our findings are in agreement with these considerations. Expression levels of the HPV encoded miRNAs described here were low, which is reasonable given that even low levels may suffice to facilitate viral replication, and that their targets may also be important for viral replication.

The significance of the predicted microRNA target sites within the E5 gene, L1 gene or LCR in the viral genome remains to be established. E5 transcripts of genital papillomaviruses are always multicistronic [53], and targeting of that particular region would affect the expression of several viral genes. Autoregulation of viral replication as shown for polyomavirus microRNAs, for example to establish latency, remains an intriguing possibility in the pathogenesis of papillomaviruses.

This paper is the first to report validated microRNAs encoded by papillomaviruses. In our approach putative viral microRNAs were sequenced and identified directly from biological material, in disease tissues from papillomavirus induced lesions, and in cancer derived cell lines, and viral microRNA expression was further shown in additional tissue samples. Reports showing the expression of viral microRNAs in human samples are rare [16]. To our knowledge, this is the first paper to use *in situ* hybridization to show the expression of viral microRNA in human tissue.

Here we have described the discovery and validation analysis of HPV encoded microRNAs using a combination of next generation sequencing, qRT-PCR and *in situ* hybridization. Altogether nine candidate microRNAs were identified. The expression of four out of five studied miRNAs was confirmed in human tissue or human epithelial cell lines harboring HPV 16. Another four candidate HPV miRNAs still await experimental validation. Biological functions of the predicted cellular target genes suggest important functions in the establishment of infection and in carcinogenesis. Viral microRNAs are also tempting as possible targets for new antiviral drugs. These findings emphasize the need for further studies on HPV miRNA functions.

## Supporting Information

Figure S1 **Schematic presentation of reads used in prediction.** Reads (red lines) were mapped to reference genome (black line). First, if the gap between mapped reads was smaller than 40 nt, the locations of reads were combined to consider the candidate pre-miRNA location. Most highly expressed reads were supposed to represent mature miRNA. Then, remaining reads were blasted to 100 nt upstream or downstream to find candidate pre-miRNA sequences.
(TIF)

Figure S2 **Visualization of candidate viral miRNA expression profiles and RNA structures.** For each predicted miRNA, its expression profiles (WIG format) from 12 sequencing libraries are shown in Integrative Genomics Viewer. Red bars present the reads mapped to the reference genome. Each row presents one library from Lib1 (first row) to Lib12 (last row). The RNA secondary structure of pre-miRNA was predicted by RNAfold, colored by base-pairing probabilities. The mature miRNA sequences are highlighted in the schematic secondary structure. A. HPV6-miR-H1; B. HPV16-miR-H1; C. HPV16-miR-H2; D. HPV16-miR-H3; E. HPV16-miR-H5; F. HPV16-miR-H6; G. HPV38-miR-H1; H. HPV45-miR-H1; I. HPV68-miR-H1.
(TIF)

Figure S3 **Visualization of reads alignment for HPV16-miR-H1/H2.** Some mapped reads of HPV16-miR-H1/H2 from sequencing library 7 (Lib7) are shown. The arrow indicates the predicted HPV16-miR-H1-1 sequence. Colorspace reads from SOLiD sequencing platform are converted to basespace. Gray or blue color depicts one or two base mismatches found in colorspace but not in basespace. Green, yellow or red color stands for one, two and three mismatches in basespace respectively.
(TIF)

Figure S4 **Prediction of HPV16-miR-H1 pre-miRNA sequence.** RNA secondary structure of pre-miRNA predicted from miRSeqNovel and VMir. The pre-miRNA from miRSeqNovel is longer because it covers the reads within 40 nt gaps, while pre-miRNA from VMir is selected from the most stable RNA structure.
(TIF)

Figure S5 **In situ hybridization for HPV 16 E1 mRNA.** To control for microRNA specificity of HPV16-miR-H1 signal, slides were hybridized under the same experimental conditions to a probe specific for HPV 16 E1 mRNA. Absence of E1 mRNA signal is shown in a CIN 1 sample. The figure fields for U6 and HPV16-miR-H1-1 are the same as in Figure 3.
(TIF)

Figure S6 **Venn Diagram of HPV16-miR-H1-1 and HPV16-miR-H2-1 targets.** HPV16-miR-H1-1 has 137 predicted targets in human genome, while HPV16-miR-H2-1 has 176. They share 15 common mRNA targets.
(TIF)

Table S1 Sequences of PCR primers as well as in situ and northern hybridization probes with their Tm values. F*, forward primer; R*, reverse primer.
(XLSX)

Table S2 393 Complete papillomavirus genomes in reference genome. Each raw present one complete papillomavirus genome with its NCBI Reference Sequence, start and end position in the reference genome and its full name.
(XLSX)

Table S3 First round prediction result on positive strand of reference genome.
(XLSX)

Table S4 First round prediction result on negative strand of reference genome.
(XLSX)

Table S5 Second round prediction result on positive strand of reference genome.
(XLSX)

Table S6 Second round prediction result on negative strand of reference genome.
(XLSX)

Table S7 Nine candidates miRNA prediction results in Table S3–6. Each raw presents one predicted miRNA with its information of strand, start location in the reference genome, pre-miRNA length, total mapped reads in pre-miRNA, RNA MFE (minimum free energy) and its papillomavirus annotation.
(XLSX)

Table S8 Complete result table of TaqMan qPCR reactions with Ct-values. No signal was obtained in runs showing empty cells. NA, not analyzed.
(XLSX)

Table S9 Target prediction of HPV16-miR-H1 and HPV16-miR-H2 in human genome. The official symbol and name of each target is shown, as well as the number of different conserved target sites. HPV16-miR-H1 has 137 conserved targets and HPV16-miR-H2 has 176 conserved targets.
(XLSX)
