# Supplementary figures and images for: Identification and Validation of Human Papillomavirus Encoded microRNAs
Source: PLoS One. 2013 Jul 30;8(7):e70202. doi: 10.1371/journal.pone.0070202 (PMC3728184; doi:10.1371/journal.pone.0070202)

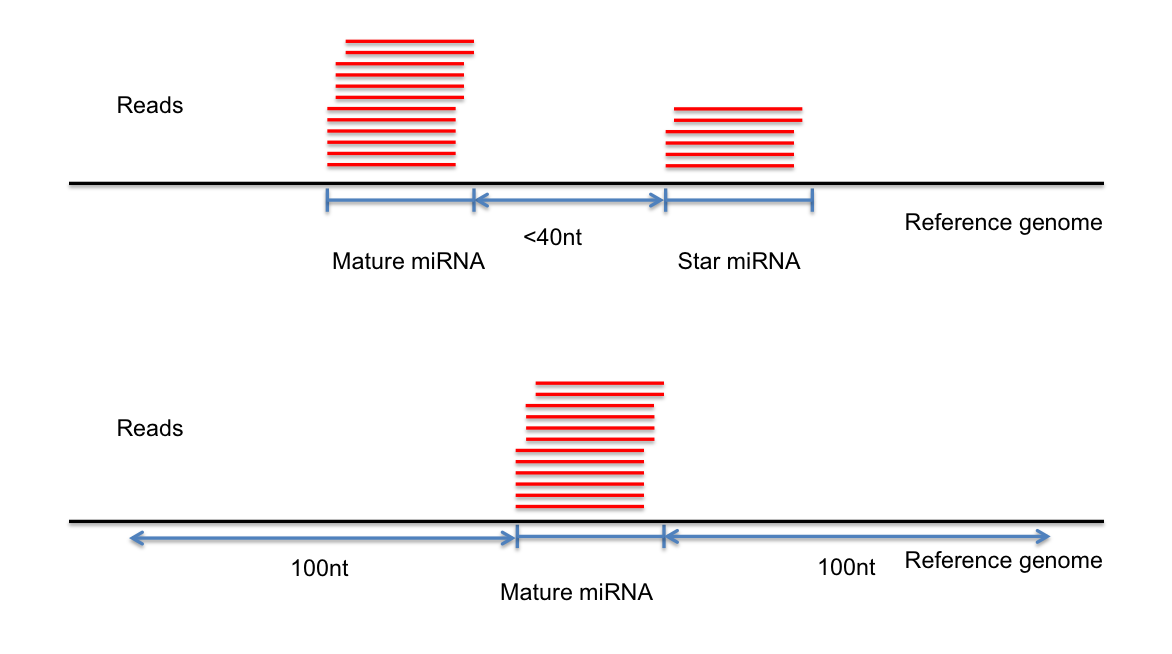

Supplement: Figure S1 — Schematic presentation of reads used in prediction. Reads (red lines) were mapped to reference genome (black line). First, if the gap between mapped reads was smaller than 40 nt, the locations of reads were combined to consider the candidate pre-miRNA location. Most highly expressed reads were supposed to represent mature miRNA. Then, remaining reads were blasted to 100 nt upstream or downstream to find candidate pre-miRNA sequences. (TIF) [file pone.0070202.s001.tif]

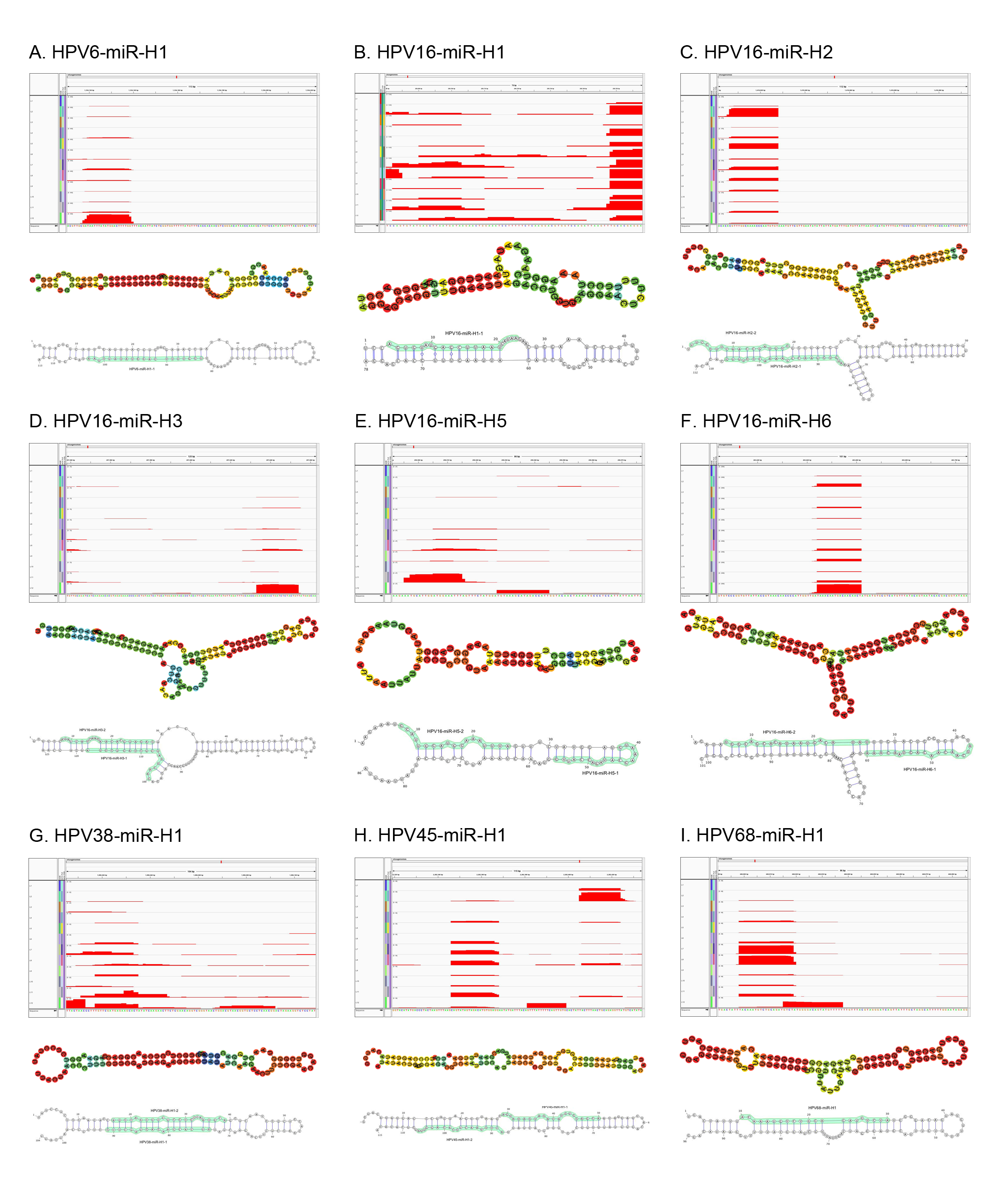

Supplement: Figure S2 — Visualization of candidate viral miRNA expression profiles and RNA structures. For each predicted miRNA, its expression profiles (WIG format) from 12 sequencing libraries are shown in Integrative Genomics Viewer. Red bars present the reads mapped to the reference genome. Each row presents one library from Lib1 (first row) to Lib12 (last row). The RNA secondary structure of pre-miRNA was predicted by RNAfold, colored by base-pairing probabilities. The mature miRNA sequences are highlighted in the schematic secondary structure. A. HPV6-miR-H1; B. HPV16-miR-H1; C. HPV16-miR-H2; D. HPV16-miR-H3; E. HPV16-miR-H5; F. HPV16-miR-H6; G. HPV38-miR-H1; H. HPV45-miR-H1; I. HPV68-miR-H1. (TIF) [file pone.0070202.s002.tif]

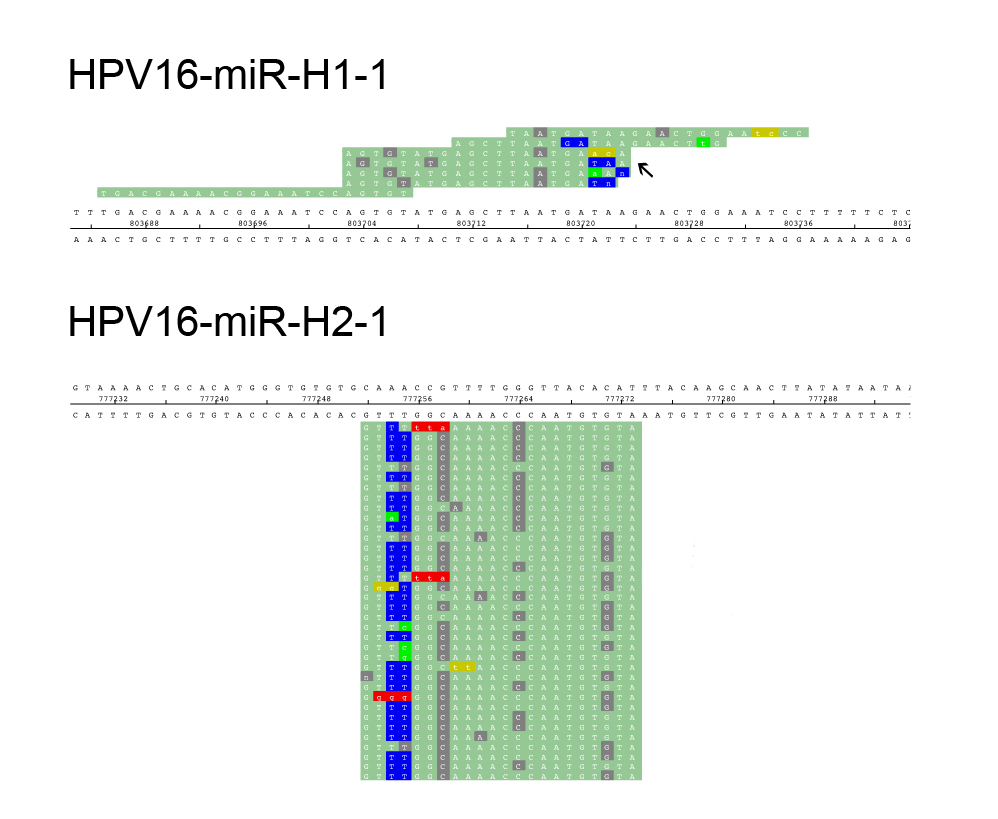

Supplement: Figure S3 — Visualization of reads alignment for HPV16-miR-H1/H2. Some mapped reads of HPV16-miR-H1/H2 from sequencing library 7 (Lib7) are shown. The arrow indicates the predicted HPV16-miR-H1-1 sequence. Colorspace reads from SOLiD sequencing platform are converted to basespace. Gray or blue color depicts one or two base mismatches found in colorspace but not in basespace. Green, yellow or red color stands for one, two and three mismatches in basespace respectively. (TIF) [file pone.0070202.s003.tif]

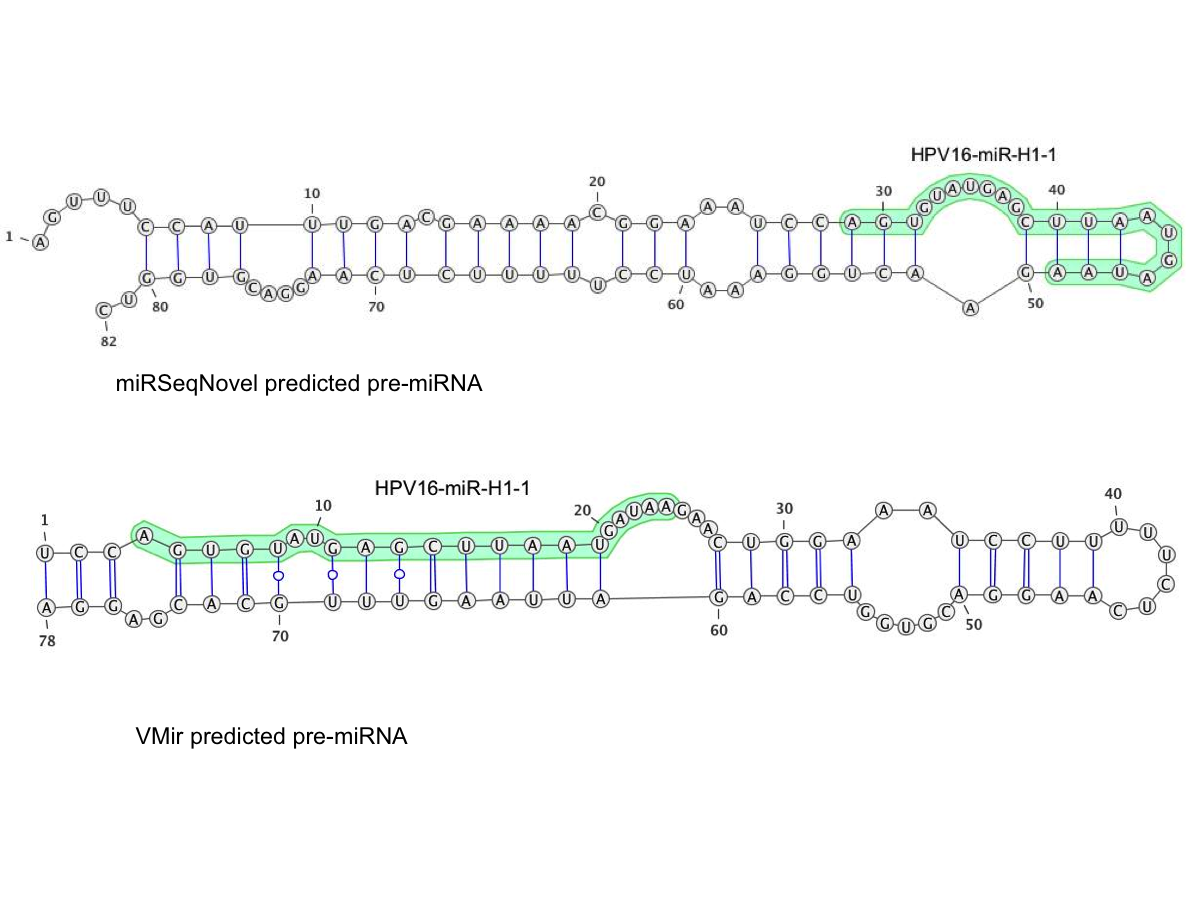

Supplement: Figure S4 — Prediction of HPV16-miR-H1 pre-miRNA sequence. RNA secondary structure of pre-miRNA predicted from miRSeqNovel and VMir. The pre-miRNA from miRSeqNovel is longer because it covers the reads within 40 nt gaps, while pre-miRNA from VMir is selected from the most stable RNA structure. (TIF) [file pone.0070202.s004.tif]

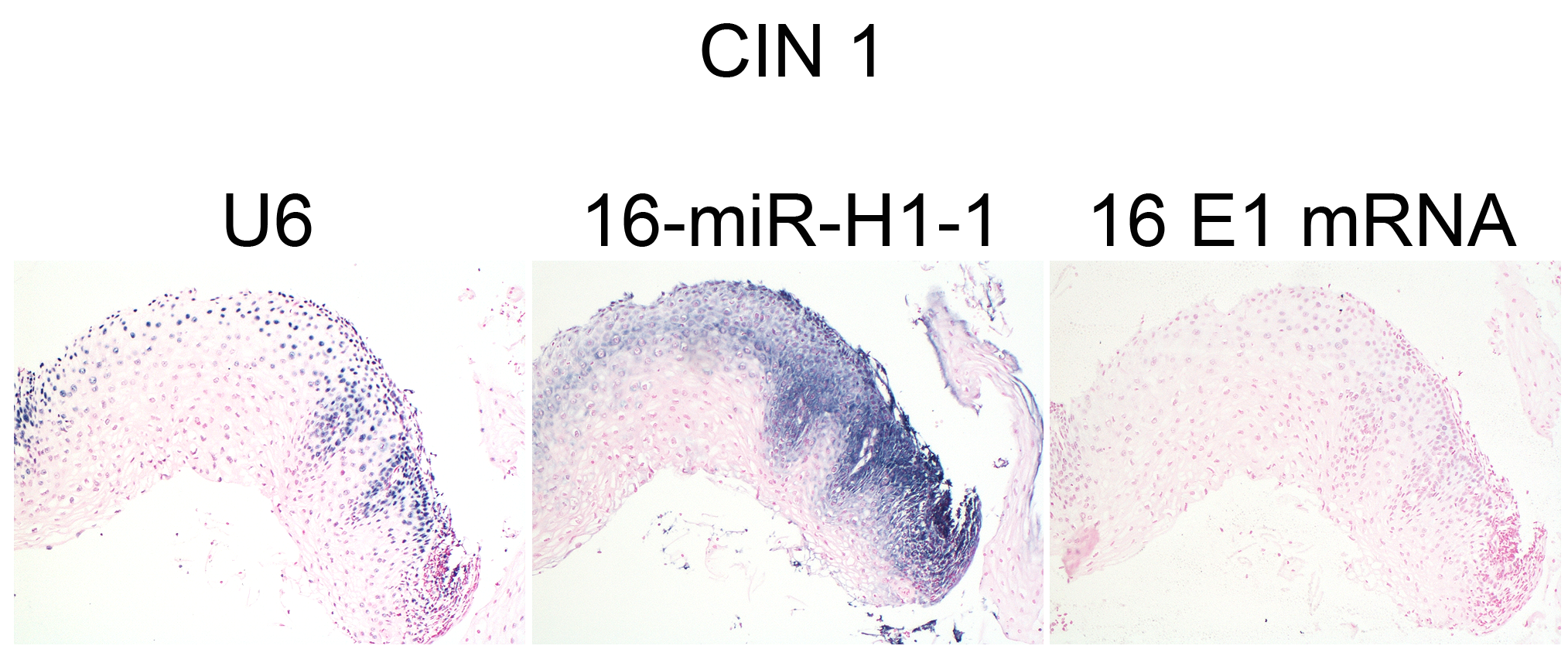

Supplement: Figure S5 — In situ hybridization for HPV 16 E1 mRNA. To control for microRNA specificity of HPV16-miR-H1 signal, slides were hybridized under the same experimental conditions to a probe specific for HPV 16 E1 mRNA. Absence of E1 mRNA signal is shown in a CIN 1 sample. The figure fields for U6 and HPV16-miR-H1-1 are the same as in Figure 3. (TIF) [file pone.0070202.s005.tif]

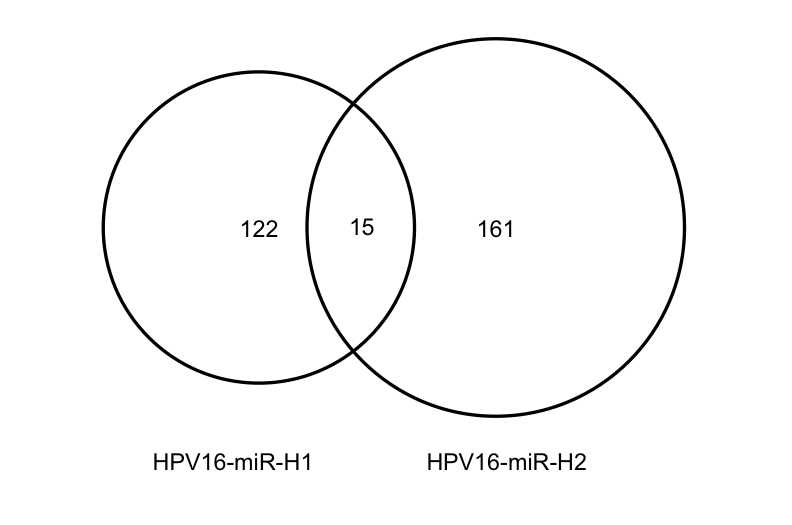

Supplement: Figure S6 — Venn Diagram of HPV16-miR-H1-1 and HPV16-miR-H2-1 targets. HPV16-miR-H1-1 has 137 predicted targets in human genome, while HPV16-miR-H2-1 has 176. They share 15 common mRNA targets. (TIF) [file pone.0070202.s006.tif]
